# VEP Abnormalities in Treatment-Naïve CIS/Early RRMS Without Prior Optic Neuritis: Clinical, Radiological, and CSF Associations

**DOI:** 10.3390/medicina62040713

**Published:** 2026-04-08

**Authors:** Furkan Sarıdaş, Rifat Özpar, Emel Oğuz Akarsu, Yasemin Dinç, Güven Özkaya, Emine Rabia Koç, Bahattin Hakyemez, Ömer Faruk Turan

**Affiliations:** 1 Department of Neurology, Faculty of Medicine, Bursa Uludağ University, 16059 Bursa, Türkiye; 2 Department of Radiology, Faculty of Medicine, Bursa Uludağ University, 16059 Bursa, Türkiye; 3 Department of Biostatistics, Faculty of Medicine, Bursa Uludağ University, 16059 Bursa, Türkiye

**Keywords:** visual evoked potential, P100 wave, treatment-naïve, early multiple sclerosis

## Abstract

*Background and Objectives:* Visual evoked potentials (VEPs) are a simple, noninvasive method for detecting subclinical visual pathway involvement in multiple sclerosis. This study investigated the frequency of VEP abnormalities and their associations with baseline clinical, radiological, and cerebrospinal fluid (CSF) features in treatment-naïve patients with clinically isolated syndrome (CIS) or early relapsing-remitting multiple sclerosis (RRMS) without prior optic neuritis. *Materials and Methods:* We retrospectively reviewed newly diagnosed, treatment-naïve CIS/early RRMS patients evaluated between January 2022 and July 2024 who underwent CSF analysis. Pattern-reversal VEPs were recorded under standardized conditions. VEP abnormalities were analyzed as any or bilateral, and associations were assessed using group comparisons and multivariable logistic regression. *Results*: In 101 patients (mean age 31.8 ± 9.7 years; 72% female; median EDSS 1.0), latency prolongation occurred in 69 (42 any,27 bilateral) and amplitude reduction in 33 (22 any, 11 bilateral). Among patients with latency prolongation, both the number of OCB bands and the IgG index were higher (bilateral *p* = 0.032; any *p* = 0.007). In multivariable analysis, male sex (*p* = 0.032) and pyramidal/brainstem-onset presentation (*p* = 0.006) were independently associated with any amplitude reduction; neither was associated with latency abnormalities. *Conclusions*: VEP abnormalities are common early in the disease, even without a history of optic neuritis. Male sex and pyramidal/brainstem-onset presentation were associated with reduced amplitude, suggesting that amplitude decrease may reflect early tissue dysfunction and may be related to adverse baseline clinical features. Associations between intrathecal immune activation and prolonged latency may indicate subclinical demyelination of the visual pathways related to inflammatory activity. Larger longitudinal studies are needed to clarify the clinical significance of VEP abnormalities in early RRMS.

## 1. Introduction

Multiple sclerosis (MS) is an immune-mediated disease of the central nervous system (CNS), characterized by chronic inflammation and demyelination, leading to progressive neurological dysfunction [1]. Subclinical CNS damage may occur before the onset of the first clinical symptoms. The first clinical episode suggestive of MS is defined as clinically isolated syndrome (CIS) in the absence of supportive clinical or paraclinical findings [2]. Long-term follow-up studies have shown that the risk of conversion from CIS to MS is relatively high [3]. Magnetic resonance imaging (MRI) and evoked potential studies can demonstrate silent lesions that do not correlate with clinical symptoms, thereby providing evidence of dissemination in space. Although significant advances have been made in the understanding of MS pathogenesis and treatment in recent years, reliable early prognostic biomarkers remain limited. The presence of oligoclonal immunoglobulin bands in the cerebrospinal fluid (CSF) and MRI findings can be used to predict disease activity and prognosis in the early stages. However, the role of environmental factors, such as vitamin D deficiency, and other biological or immunological biomarkers, remains controversial [4,5].

In early MS, inflammatory demyelination and neuroaxonal injury may affect functional pathways even before permanent structural damage becomes clinically evident. In the visual system, demyelination disrupts saltatory conduction and slows impulse transmission, whereas axonal loss reduces the number of conducting fibers. These pathological changes may be partially counterbalanced by remyelination and functional reorganization; therefore, electrophysiological measures may serve as functional markers of both tissue injury and repair. In this context, VEP abnormalities can be interpreted not only as indicators of visual pathway involvement but also as reflections of broader mechanisms underlying early MS pathology [6,7].

Visual evoked potential (VEP) testing, used to detect optic neuritis (ON) and visual system dysfunction in MS, has proven a valuable outcome measure in clinical trials. It also provides a sensitive tool for assessing longitudinal changes in MS associated with underlying neuropathology. Prolongation of the latency of its main component, the P100 waveform peak, generally reflects myelin integrity in the context of demyelination and the capacity for repair in remyelination processes [8]. The amplitude of the N75–P100 waveform reflects the number of functional fibers within the visual pathway and serves as an indicator of axonal dysfunction [9,10,11]. VEP is considered one of the most sensitive methods for evaluating functional remyelination in MS [12,13].

This study aims to investigate the relationship between VEP findings and baseline clinical, radiological, and laboratory features in patients with MS and CIS, including variables previously associated with less favorable disease course.

## 2. Material and Methods

### 2.1. Patient Selection

**Figure 1:**
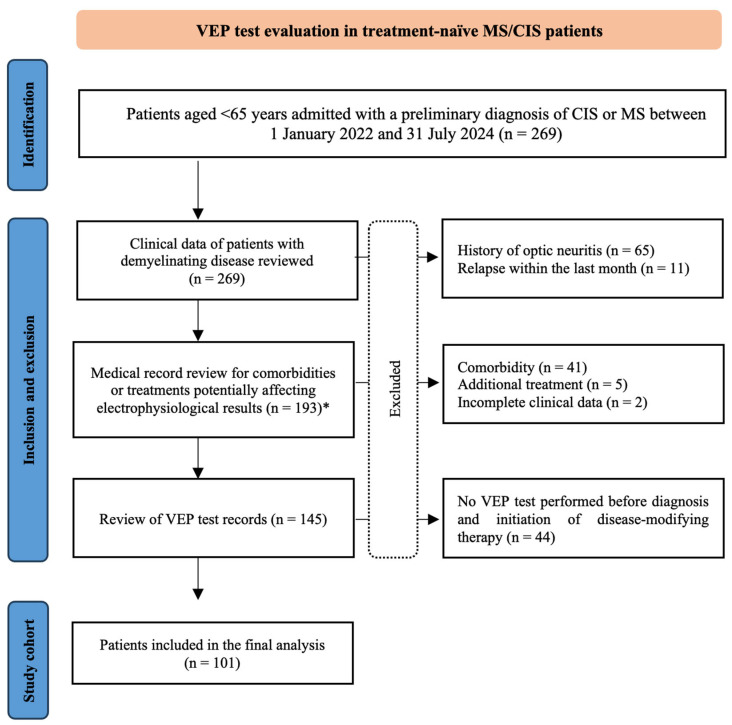
Patient selection flowchart after inclusion and exclusion criteria. * Number of excluded patients: fourteen patients with diabetes mellitus, seven with rheumatological diseases, three with epilepsy, twelve with thyroid disorders, four with malignancies, one with cerebrovascular disease, and five with statin use.

The medical records of 270 patients admitted to our center between 1 January 2022, and 31 July 2024, with a preliminary diagnosis of CNS demyelinating disease, cerebrospinal fluid examination, and MS or CIS diagnosis according to the 2017 revised McDonald criteria, were retrospectively reviewed. Patients with a history of optic neuritis at any time, those who experienced any demyelinating relapse within the previous month, those with potentially confounding comorbid conditions or concomitant medication use that could affect optic nerve conduction, those who had received any disease-modifying therapy prior to VEP testing, and those with insufficient clinical data were excluded from the study. Potentially confounding comorbidities and treatments were identified through retrospective review of the electronic medical records using the same predefined exclusion framework for all screened cases (Figure 1). These included conditions or treatments considered potentially relevant to electrophysiological interpretation, such as diabetes mellitus, rheumatologic diseases, epilepsy, thyroid disorders, malignancy, cerebrovascular disease, and statin use. After applying the exclusion criteria, 101 newly diagnosed, treatment-naïve patients were included in the study. See Figure 1 for details on patient selection. The local ethics committee has approved the study (number 2024-13/2). The ethics committee waived the requirement for informed consent due to the study’s retrospective design. All procedures were performed in accordance with the relevant guidelines and regulations.

### 2.2. Clinical, Radiological, Laboratory Data, and VEP Assessment

Demographic characteristics, first presenting symptom, and Expanded Disability Status Scale (EDSS) scores were recorded. Laboratory data included CSF analysis results (chloride, glucose, cell count, oligoclonal band type/number, IgG index) as well as baseline serum vitamin B12 and 25-hydroxyvitamin D levels obtained prior to any supplementation.

The localization of demyelinating lesions was evaluated on cranial MRI (cortical/juxtacortical, periventricular, infratentorial, corpus callosum) and, when available, spinal MRI (cervical, thoracic). MRI scans performed both at our center and at external institutions were reviewed and included in the analysis. All radiological assessments were performed by consensus between two expert neuroradiologists.

Cognitive and functional assessments, including the Symbol Digit Modalities Test (SDMT), Montreal Cognitive Assessment (MoCA), Nine-Hole Peg Test (9HPT), and Timed 25-Foot Walk (T25FW), were administered by the same psychologist in accordance with standardized testing procedures.

**Figure 2:**
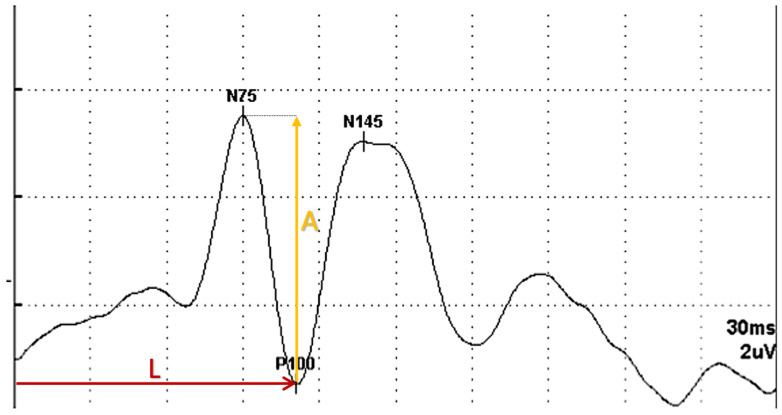
Sample test output showing the P100 component. L (red): latency (ms). A (yellow): amplitude (µV).

Visual evoked potentials were administered and recorded by the same technician under standardized environmental conditions, using pattern-reversal stimulation with an 8 × 8 checkerboard pattern and the same device and software, with each trace averaged from at least 100 sweeps and optimal refractive correction when applicable. All recordings were reviewed for technical adequacy, and traces with evident artifact were excluded from analysis. A P100 latency longer than 118 ms and an N75–P100 amplitude lower than 5.0 µV were considered abnormal, based on the normative reference values used in our electrophysiology laboratory for the same device and stimulation protocol. VEP results were classified as any abnormality when a reduction in amplitude or prolongation of latency was present on at least one side, and as bilateral abnormalities when abnormalities were detected on both sides (see Figure 2 for an example of a VEP recording).

Clinical, laboratory, radiological, cognitive, and electrophysiological data were recorded following retrospective review of patient medical records. Clinical and electrophysiological parameters were evaluated and agreed upon by three neurologists, one of whom was a neurophysiologist. Clinical, laboratory, and radiological findings were compared between the groups.

### 2.3. Statistical Analysis

The Shapiro–Wilk test was used to assess the normality of data distribution. Descriptive statistics were presented as mean ± standard deviation or median (minimum–maximum) for quantitative variables, and as frequency and percentage for qualitative variables. Kruskal–Wallis and Mann–Whitney U tests were used for nonnormally distributed data. Categorical variables were analyzed using the Pearson Chi-square test, Fisher–Freeman–Halton test, or Fisher’s exact test, as appropriate. When multiple comparisons were performed, a correction for multiple testing was applied using the Bonferroni method. The relationships between variables were evaluated using Pearson or Spearman correlation coefficients, depending on data distribution. VEP parameters were analyzed both categorically and as continuous variables. Categorical analyses were based on predefined abnormality thresholds to facilitate clinically interpretable group comparisons, whereas continuous analyses were used to preserve measurement variability and reduce information loss associated with dichotomization. These complementary approaches were intended to provide both clinically relevant classification and a more sensitive assessment of the robustness of observed associations. Both univariate and multivariable binary logistic regression analyses were performed, and odds ratios (ORs) with 95% confidence intervals (CIs) were reported. The level of statistical significance was set at α = 0.05. Because of the retrospective design, not all variables were available for every patient. Analyses were therefore performed using available case data for each variable, and the number of patients included in each analysis is reported where relevant. All statistical analyses were performed using IBM SPSS Statistics for Windows, version 28.0 (IBM Corp., Armonk, NY, USA). Graphs were generated using Jamovi (The Jamovi Project, 2025, Sydney, Australia).

## 3. Results

The cohort included 101 patients (73 females, 28 males; 87 RRMS, 14 CIS) with a mean age of 31.8 ± 9.69 years. The most frequent disease onset symptoms were sensory (56.4%), followed by pyramidal (30.7%), brainstem (23.8%), and other symptoms (22.8%). The median EDSS score was 1 (range: 0–4.5). A total of 82 patients completed the Nine-Hole Peg Test (9HPT) (mean time: 22.74 ± 8.8 s for the right hand and 23.32 ± 6.18 s for the left hand), 80 completed the Timed 25-Foot Walk (T25FW) (mean time: 7.08 ± 1.54 s), 81 completed the Symbol Digit Modalities Test (SDMT) (mean score: 35.33 ± 12.3), and 71 completed the Montreal Cognitive Assessment (MoCA) (mean score: 24 ± 3.8).

CSF protein levels were available for all patients; CSF cell count and chloride levels were available for all except one patient; the CSF/serum glucose ratio was available in 88 patients; advanced immunological CSF analyses were available in 87 patients; and opening pressure measurements were available in 79 patients. The mean CSF protein level was 31.8 ± 12.5 mg/dL (13.8–82.2). The mean CSF/serum chloride ratio was 1.17 ± 0.03, and the mean CSF/serum glucose ratio was 0.64 ± 0.12. The mean CSF cell count was 4.18 cells/µL (0–105). Opening pressure measurements were available in 79 patients, with a mean value of 15.1 ± 5.92 cm H_2_O. Advanced immunological CSF analyses were available in 87 patients. Oligoclonal band (OCB) analysis showed type 1 in 16 patients, type 2 in 55, type 3 in 15, and type 4 in 1 patient. The mean number of OCBs was 12.6 ± 7.02 (2–27), and the mean IgG index was 0.82 ± 0.48 (0.34–2.77). Vitamin B12 levels in three patients and 25-hydroxyvitamin D levels in ten patients were excluded from analysis because the patients were receiving replacement therapy or the tests had not been performed. The mean serum 25-hydroxyvitamin D level was 12.7 ± 11.2 µg/L, and the mean vitamin B12 level was 316 ± 142 ng/L.

Cranial MRI was performed in 96 patients, cervical MRI in 92, and thoracic MRI in 38. Lesion frequencies were calculated within the imaged subgroups: periventricular (91.7%), cortical/juxtacortical (64.6%), infratentorial (42.7%), and corpus callosum (34.7%) on cranial MRI; cervical spinal cord involvement (60.9%) on cervical MRI; and thoracic spinal cord involvement (47.4%) on thoracic MRI.

Any latency prolongation (whether unilateral or bilateral) was observed in 42 patients (41.6%), including bilateral latency prolongation in 27 patients (26.7%). Any amplitude reduction was detected in 22 patients (22%), including bilateral amplitude reduction in 11 patients (11%). Among the clinical variables examined, amplitude reduction showed the clearest associations. Any amplitude reduction was associated with male sex (*p* = 0.015) and poorer dominant hand performance (*p* = 0.047), while brainstem symptoms at onset were associated with both amplitude reduction and latency prolongation in any and bilateral analyses (Table 1). No significant associations were observed for other clinical characteristics or lesion localizations.

Although OCB counts (any: *p* = 0.045 vs. 0.485; bilateral: *p* = 0.034 vs. 0.612) and the CSF/serum chloride ratio (any: *p* = 0.042 vs. 0.199) differed according to the presence of amplitude reduction, these differences were not significant when analyzed categorically. Regarding latency prolongation, although any latency prolongation was more frequent in patients with elevated CSF protein levels (>40 mg/dL) (*p* = 0.046), CSF protein level itself was not significantly associated with latency changes (*p* = 0.573). In contrast, any latency prolongation was significantly associated with a higher mean IgG index (0.71 vs. 0.97, *p* = 0.007), elevated IgG index (>0.7) (36% vs. 61.1%, *p* = 0.029), higher mean OCB count (11.19 vs. 16.67, *p* = 0.032), and elevated OCB band count (>12 bands) (*p* = 0.026). Low vitamin D levels (<20 µg/L) were associated with bilateral amplitude reduction (*p* = 0.030); however, mean serum vitamin D levels did not differ according to the presence of amplitude reduction (*p* = 0.207) (Table 2).

**Figure 3:**
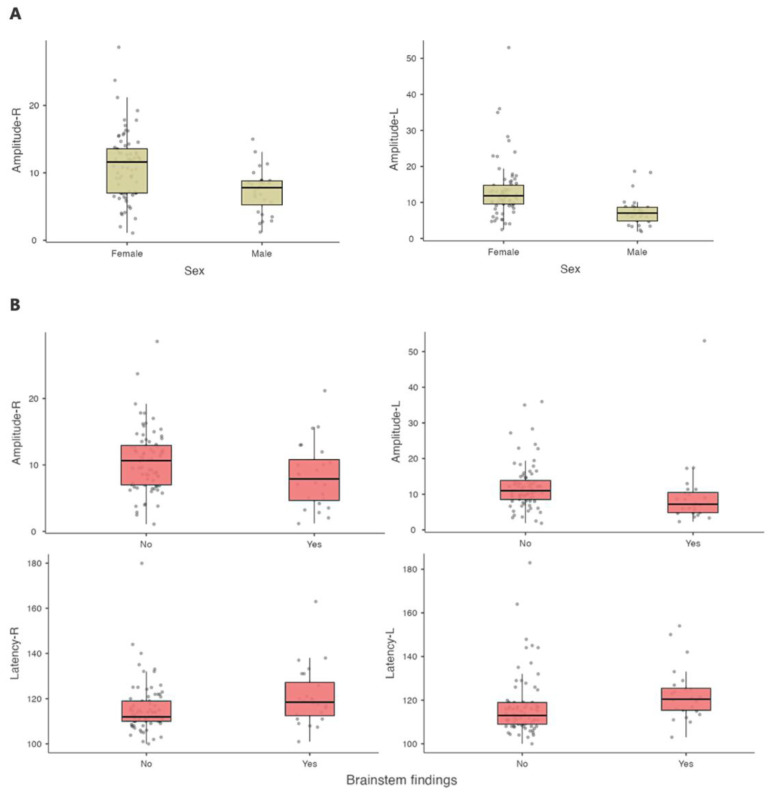
VEP differences by sex and brainstem-onset presentation at disease onset. (**A**)—Females showed higher right and left amplitude values compared to males. Mean right/left amplitudes were 11.1 ± 4.96 and 13.2 ± 7.94 in females, and 7.32 ± 3.26 and 7.56 ± 4.12 in males, respectively. These differences were statistically significant for both sides (both *p* < 0.001). (**B**)—Patients with a brainstem-onset presentation had significantly lower amplitudes and longer latencies than those without brainstem-onset at disease onset. Mean right-sided amplitude was 8.36 ± 4.95 versus 10.6 ± 4.72 (*p* = 0.048), and mean left-sided amplitude was 9.83 ± 10.0 versus 12.2 ± 6.50 (*p* = 0.003). Mean right-sided latency was significantly higher in patients with brainstem involvement (121 ± 13.1 ms vs. 116 ± 11.3 ms, *p* = 0.031), as was mean left-sided latency (123 ± 12.1 ms vs. 117 ± 13.8 ms, *p* = 0.003).

**Figure 4:**
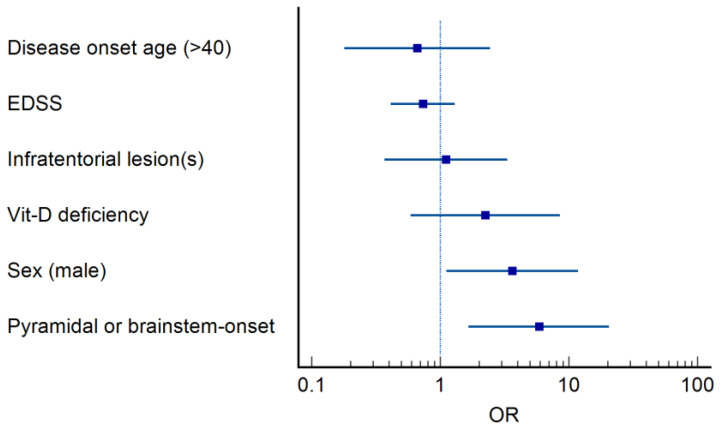
Forest plot of independent predictors of any amplitude reduction in multivariable logistic regression. Male sex (OR = 3.635, 95% CI: 1.119–11.808) and pyramidal or brainstem onset (OR = 5.865, 95% CI: 1.660–20.513) were independently associated with any amplitude reduction.

When VEP P100 measurements were analyzed as continuous variables, independent of predefined cut-off values, male sex, brainstem-onset clinical presentation, and poorer dominant hand performance remained significantly associated with VEP abnormalities. (Table 1, Figure 3, and Appendix A). In multivariable analysis, male sex (OR = 3.635, 95% CI: 1.119–11.808, *p* = 0.032) and initial pyramidal or brainstem involvement (OR = 5.835, 95% CI: 1.660–20.513, *p* = 0.006) emerged as independent predictors of any amplitude reduction. By contrast, no independent predictors were identified for latency prolongation. Although the model for bilateral amplitude reduction reached overall statistical significance, no individual variable remained independently associated; given the small number of bilateral events, this result should be interpreted cautiously and may reflect limited model stability. Detailed results are presented in Table 3 and Figure 4.

## 4. Discussion

In this study, we evaluated the frequency of visual evoked potential abnormalities in treatment-naïve patients with clinically isolated syndrome and early multiple sclerosis without a history of optic neuritis, and their associations with clinical, radiological, and CSF findings. Our results demonstrate that VEP abnormalities are highly prevalent even in the early stages of the disease and are associated with several adverse baseline clinical features. VEP is a simple, noninvasive, and time-efficient technique that can detect subclinical visual pathway involvement and reflect alterations in conduction and tissue integrity. In this context, it may serve as a useful supportive measure in the assessment of early multiple sclerosis, although its prognostic significance requires confirmation in longitudinal studies [14].

In our cohort, latency prolongation was observed in approximately two-fifths of patients and reduced amplitude in about one-fifth, findings consistent with previous studies reporting that subclinical visual pathway involvement is common in CIS/MS patients, even in the absence of a history of optic neuritis [15,16]. This finding suggests that VEP may be capable of detecting “clinically silent” demyelinating involvement in the early stages of the disease [7,17].

Male sex, severity of the first demyelinating event, time to the second event, older age at onset, presence of pyramidal symptoms, multifocal onset, or lesion localization are considered prognostic factors associated with the future clinical course of multiple sclerosis [18,19,20,21,22]. One of the most notable findings of our study is the consistent association between male sex and pyramidal or brainstem onset of the first clinical event with VEP abnormalities. In multivariable analyses, male sex was associated with approximately a 3.6-fold increased risk, and pyramidal or brainstem onset with approximately a 5.8-fold increased risk, particularly for any amplitude reduction. These findings suggest that early amplitude abnormalities may be associated with baseline clinical features previously linked to a less favorable disease course. The existing literature linking male sex to poorer long-term outcomes in multiple sclerosis further supports the clinical significance of this observation [23,24,25]. In addition, normative studies have reported that VEP responses may exhibit slight sex-related differences, with women showing somewhat shorter P100 latencies and slightly higher amplitudes compared to men [26,27,28]. However, in our cohort, the much more pronounced reduction in amplitude among male patients and its more frequent occurrence within the pathological range (<5 µV) suggest that this finding cannot be explained solely by normative sex-related differences and may reflect a clinically meaningful phenomenon.

Extensive cohort studies have long demonstrated that clinical onset involving pyramidal and/or brainstem systems is associated with more rapid disability accumulation and poorer long-term outcomes compared with sensory or optic onset [29,30]. The finding that reduced P100 amplitude was associated with baseline clinical characteristics previously linked to less favorable disease course supports the notion that amplitude abnormalities may represent a clinically relevant electrophysiological correlate of adverse baseline disease features. However, this association should not be interpreted as direct evidence of a specific underlying mechanism. In contrast, no independent association was identified between any VEP abnormality and other potential adverse prognostic factors, including older age at disease onset, EDSS score, presence of infratentorial lesions, or vitamin D deficiency. The lack of an independent association between vitamin D deficiency and VEP abnormalities should be interpreted cautiously. Although vitamin D has been implicated in MS susceptibility and disease activity, the absence of a stronger relationship in our cohort may be related to the limited sample size, incomplete availability of vitamin D measurements, exclusion of patients receiving replacement therapy, reliance on a single baseline assessment, and possible seasonal variability. Overall, these findings suggest that some prognostic factors may exert weaker or more indirect effects on electrophysiological outcomes. Recent evidence emphasizing the multifactorial nature of prognostic determinants in multiple sclerosis and demonstrating that the effects of specific variables may diminish in multivariable models supports this interpretation [31,32,33]. From a pathophysiological perspective, prolongation of VEP latency is generally interpreted in relation to slowed conduction and demyelinating or inflammatory processes. Beyond demyelination-related conduction slowing, neurotrophin-mediated central nervous system myelination, particularly involving BDNF, may provide an additional biological framework for interpreting the present electrophysiological findings [34]. This raises the possibility that VEP changes may reflect not only tissue injury but also processes related to endogenous repair. In contrast, reduction in VEP amplitude may reflect several partly overlapping mechanisms, including axonal damage, impaired conduction, reduced neural response synchrony, and measurement-related variability. Within this framework, the association between amplitude reduction and adverse baseline clinical features may indicate greater early tissue dysfunction or injury; however, this interpretation should remain cautious, as amplitude changes are not specific to neuroaxonal damage alone and cannot establish mechanism in the present study [35].

Intrathecal IgG synthesis (elevated IgG index and/or oligoclonal band positivity) is emphasized in recent studies as a biomarker of inflammatory activity in multiple sclerosis. In our subgroup analyses, patients with prolonged P100 latency showed higher CSF protein levels, higher IgG index values, and greater oligoclonal band burden, raising the possibility of an association between intrathecal immune activation and electrophysiological impairment of the visual pathways [36]. However, these findings should be interpreted cautiously, as some associations were derived from subgroup analyses with incomplete data availability and were attenuated when VEP parameters were analyzed as continuous variables. Accordingly, these results should be regarded as exploratory and hypothesis-generating rather than as robust mechanistic evidence.

With respect to functional outcomes, the weak but statistically significant association between upper extremity function and reduced amplitude represents a supportive finding suggesting that visual pathway involvement may coexist with more widespread functional impairment; however, the clinical relevance of this association should be confirmed in larger cohorts. Similarly, previous studies have reported that evoked potential abnormalities may correlate with higher EDSS scores and may carry prognostic value [37]. Although an association between VEP and cognitive performance has been reported, the specific mechanisms underlying this relationship remain unclear [17,38,39,40]. However, the cross-sectional design of our study and the early-stage, treatment-naïve nature of our sample may have limited our ability to detect associations in domains such as cognitive performance and lesion localization.

### Strengths and Limitations

A significant strength of this study is the evaluation of VEP abnormalities in treatment-naïve CIS and early RRMS patients without a history of optic neuritis, allowing assessment at a very early disease stage while minimizing the confounding effects of both disease-modifying treatments and clinically overt optic neuritis. The analysis of VEP parameters, not only as dichotomous (normal/abnormal) but also as continuous variables (P100 latency and amplitude), enabled a more sensitive assessment. Moreover, the integrated evaluation of clinical, MRI, and CSF data allowed for a multidimensional interpretation of potential biological mechanisms. The independent associations of male sex and pyramidal or brainstem onset with amplitude pathology in multivariable models represent hypothesis-generating findings that support the potential relevance of VEP in the multidimensional assessment of early disease. Nevertheless, the cross-sectional design precluded direct assessment of long-term disease progression, conversion, or disability accumulation. Therefore, although the observed VEP abnormalities were associated with several adverse baseline clinical features, it remains uncertain whether these electrophysiological findings translate into differences in long-term clinical outcomes. In addition, the relatively small sample size and low number of events in some subgroup outcomes, particularly bilateral amplitude reduction, may have limited the stability of the multivariable logistic regression models and increased the risk of overfitting. Therefore, these regression analyses should be interpreted as exploratory and hypothesis-generating.

Future studies should be designed prospectively and longitudinally to validate these cross-sectional findings and to investigate the temporal dynamics of VEP changes in relation to PIRA, EDSS progression, and functional outcomes. In particular, multimodal approaches integrating standardized VEP protocols with OCT-derived retinal measures and advanced quantitative MRI markers of myelin integrity may provide a more comprehensive understanding of early tissue injury and repair in MS. Such combined approaches will be important for clarifying the biological and prognostic significance of VEP abnormalities in early disease.

## 5. Conclusions

This study demonstrates that VEP abnormalities are common in treatment-naïve CIS and early RRMS patients, even in the absence of a history of clinical optic neuritis, and can be detected at a very early stage of the disease. Male sex and pyramidal or brainstem involvement at disease onset were independently associated with any P100 amplitude reduction. These findings suggest that amplitude pathology may represent a functional electrophysiological marker of early tissue dysfunction and may be associated with adverse baseline clinical features. In addition, the association between intrathecal immune activation markers and prolonged latency suggests that subclinical demyelination of the visual pathways may be related to inflammatory activity. However, the cross-sectional design precludes conclusions regarding the independent long-term prognostic value of these findings. Further studies with larger sample sizes and longitudinal follow-up are warranted to clarify the utility of VEP as a supportive biomarker for early risk stratification and disease monitoring in multiple sclerosis.

## Figures and Tables

**Table 1:** Differences in demographic characteristics, clinical findings, cognitive performance, and extremity function assessments according to VEP test pathologies.

		Amplitude					Latency					** **
		Normal	Reduction, Any	*p*	Bilateral Reduction	*p*	Normal	Prolongation, Any	*p*	Bilateral Prolongation	*p*	Total
Age		31.71 (9.84) 31 (17–54)	32.5 (9.44), 33 (19–49)	0.748	31.91 (9.15) 31 (20–45)	0.991	31.86 (9.51) 31 (18–51)	31.71 (10.05) 31 (17–54)	0.852	32.26 (9.49) 32 (17–54)	0.776	31.8 (9.69) 31 (17–54)
Sex	Female	62 (84.9%)	11 (15.1%)	**0.015**	6 (8.3%)	0.283	44 (74.6%)	29 (69%)	0.653	19 (26%)	0.796	14 (13.9%)
	Male	17 (60.7%)	11 (39.3%)		5 (17.9%)		15 (53.6%)	13 (46.4%)		8 (28.6%)		87 (27.7%)
Disease onset age	>40	21 (26.9%)	5 (22.7%)	0.789	3 (11.5%)	1.000	15 (25.4%)	11 (26.2%)	1.000	7 (26.9%)	1.000	26 (25.7%)
	<40	57 (73.1%)	17 (77.3%)		8 (10.8%)		44 (58.7%)	31 (41.3%)		20 (26.7%)		
Diagnosis	CIS	13 (17.3%)	1 (3.8%)	1.000	2 (15.4%)	0.633	11 (18.6%)	3 (7.1%)	0.145	1 (7.1%)	0.105	14 (13.9%)
	RRMS	62 (82.7%)	25 (96.2%)		9 (10.3%)		48 (55.2%)	39 (44.8%)		26 (29.9%)		87 (86.1%)
Onset symptoms	Motor	24 (30.4%)	7 (31.8%)	1.000	1 (9.1%)	0.166	20 (33.9%)	11 (26.2%)	0.513	7 (22.6%)	0.630	31 (30.7%)
	Sensory	49 (62%)	8 (36.4%)	0.051	4 (36.4%)	0.206	36 (61%)	21 (50%)	0.312	13 (22.8)	0.367	57 (56.4%)
	Brainstem	13 (16.5%)	11 (50%)	** 0.002**	6 (54.5%)	**0.021**	9 (15.3%)	15 (35.7%)	**0.032**	12 (44.4%)	**0.005**	24 (23.8%)
	Other	21 (26.6%)	3 (13.3%)	0.271	1 (9.1%)	0.449	11 (18.6%)	12 (28.6%)	0.336	7 (30.4%)	0.789	23 (22.8%)
	Motor or BS	**31 (39.7%)**	** 17 (77.3%)**	** 0.003**	7 (14.6%)	0.345	27 (45.8%)	22 (52.4%)	0.549	16 (32.7%)	0.261	49 (48.5%)
EDSS		1.26 (1.12), 1 (0–4.5)	1.25 (0.96), 1 (0–3.5)	0.858	1.23 (1.17) 1 (0–3.5)	0.926	1.4 (1.11) 1 (0–4.5)	1.06 (1.02) 1 (0–3.5)	0.140	1.22 (1.08) 1 (0–3.5)	0.926	1.26 (1.08), 1 (0–4.5)
Baseline EDSS ≥3		7 (9%)	2 (9.1%)	1.000	2 (18.2%)	0.257	7 (11.9%)	2 (4.8%)	0.299	25 (27.2%)	1.000	9 (8.9%)
9HPT-Right		21.61 (5.78), 21 (12–42)	27.47 (15.09), 23 (15–80)	**0.047**	29.88 (20.48) 23 (19–80)	0.913	21.75 (5.8) 21 (12–42)	23.89 (11.3) 21 (15–80)	0.798	1.22 (1.08) 1 (0–3.5) 24.46 (13.24) 21 (15–80)	0.913	22.74 (8.8), 21 (12–80)
9HPT-Left		23.13 (6.32), 21 (15–48)	24.47 (5.66), 23 (17–36)	0.233	24.38 (4.17) 24.5 (18–30)	0.539	23.16 (6.43) 21 (15–45)	23.53 (5.94) 23 (16–48	0.517	23.77 (6.54) 22.5 (16–48)	0.539	23.33 (6.18), 22.5 (15–48)
T25FW		7.1 (1.52), 7 (4.5–11)	6.97 (1.71), 7 (4.5–10.5)	0.661	6.75 (1.85) 7 (4.5–10)	0.073	7.28 (1.47) 7 (4.5–11)	6.82 (1.62) 7 (4.5–10.5)	0.179	6.64 (1.69) 6.5 (4.5–10.5)	0.073	7.08 (1.54), 7 (4.5–11)
SDMT		35.3 (12.08), 36 (3–57)	34.59 (13.16), 32 (14–61)	0.746	37.78 (13.94) 32 (22–61)	0.297	36.55 (11.88) 38 (3–56)	33.89 (12.72) 32 (14–61)	0.236	33.73 (12.06) 31 (16–61)	0.297	35.33 (12.27), 36 (3–61)
MoCA		23.82 (4.06), 25 (14–30)	24.67 (2.66), 25 (19–30)	0.757	25.67 (2.94) 26 (22–30)	0.524	24 (4.08) 25 (14–30)	24 (3.46) 25 (16–30)	0.746	23.68 (3.63) 24.5 (16–29)	0.524	24 (3.77), 25 (14–30)
MRI-Lesion(s) localization	Periventricular	67 (91.8%)	20 (90.9%)	1.000	9 (81.8%)	0.231	51 (91.1%)	37 (92.5%)	1.000	24 (92.3%)	1.000	88 (91.7%)
	Cortical/juxtacortical	49 (67.1%)	13 (59.1%)	0.610	7 (63.6%)	1.000	39 (69.6%)	23 (57.5%)	0.280	17 (65.4%)	0.920	62 (64.6%)
	Corpus callosum	24 (32.9%)	9 (42.9%)	0.442	3 (27.3%)	0.741	17 (30.4%)	16 (41%)	0.381	11 (44%)	0.329	33 (34.7%)
	Infratentorial	29 (39.7%)	11 (50%)	0.463	5 (45.5%)	1.000	22 (39.3%)	19 (47.5%)	0.531	15 (57.7%)	0.070	41 (42.7%)
	Cervical	44 (63.8%)	12 (54.5%)	0.460	4 (36.4%)	0.098	30 (54.5%)	26 (70.3%)	0.191	17 (70.8%)	0.332	56 (60.9%)
	Thoracic	10 (38.5%)	8 (66.7%)	0.164	5 (71.4%)	0.222	8 (36.4%)	10 (62.5%)	0.188	7 (63.6%)	0.288	18 (47.4%)

Sample sizes for each variable were as follows: 9HPT (n = 82), T25FW (n = 80), SDMT (n = 81), MoCA (n = 71), MRI (n = 96), and amplitude (n = 100). All remaining variables were available for the entire cohort. Data are presented as n (%), mean (SD), or median (min–max). Statistical significance was defined as *p* < 0.05 and is highlighted in bold. VEP measurements were evaluated both as continuous (parametric) variables and as categorical variables based on predefined cut-off values indicating prolonged latency or reduced amplitude. Variables that were statistically significant in both analyses are highlighted. (see Appendix A). Abbreviations: EDSS: Expanded Disability Status Scale; 9HPT: Nine-Hole Peg Test; T25FW: Timed 25-Foot Walk; SDMT: Symbol Digit Modalities Test; MoCA: Montreal Cognitive Assessment; MRI: Magnetic Resonance Imaging.

**Table 2:** Differences in cerebrospinal fluid and vitamin levels according to VEP test.

Laboratory Results		Amplitude					Latency					Total
		Normal	Reduction, Any	*p*	Bilateral Reduction	*p*	Normal	Prolongation, Any	*p*	Bilateral Prolongation	*p*	
COP, cmH_2_O		14.95 (6.07) 14 (4–35)	15.76 (5.44) 16 (6–28)	0.406	16.64 (6.98) 16 (6–28)	0.406	14.76 (5.08) 15 (6–28)	15.42 (6.82) 14 (4–35)	0.900	14.63 (7.37) 14 (4–35)	0.257	15.06 (5.92) 14.5 (4–35)
COP, >20 cmH_2_O		8 (14.3%)	3 (14.3%)	1.000	3 (27.3%)	0.187	5 (11.9%)	6 (16.7%)	0.746	3 (27.3%)	1.000	11 (14.1%)
Protein, mg/dL		31.03 (11.95) 27 (13.8–82.2)	34.82 (14.41) 30.25 (16.4–69)	0.298	34.63 (18) 27 (16.4–69)	0.982	30.66 (12.12) 27 (13.8–82.2)	33.5 (12.96) 28.9 (17–74)	0.308	31.54 (10.88) 27 (17–56)	0.972	31.84 (12.49) 27.2 (13.8–82.2)
Protein, >40 mg/dL		15 (19.2%)	6 (27.3%)	0.393	3 (27.3%)	0.695	8 (13.6%)	13 (31%)	**0.046**	7 (33.3%)	0.580	21 (20.8%)
Cell count, /mm^3^		5.1 (15.55) 0 (0–105)	1.14 (3) 0 (0–10)	0.090	2.27 (4) 0 (0–10)	0.893	4.36 (14.28) 0 (0–105)	3.93 (13.28) 0 (0–84)	0.573	1.44 (3.32) 0 (0–11)	0.188	4.18 (13.8) 0 (0–105)
Cell, >10/mm^3^		14 (18.2%)	1 (4.5%)	0.179	1 (9.1%)	1.000	9 (15.5%)	6 (14.3%)	1.000	2 (13.3%)	0.343	15 (14.9%)
Chlorine R		1.17 (0.03) 1.17 (1.09–1.29)	1.18 (0.03) 1.19 (1.14–1.28)	**0.042**	1.18 (0.04) 1.18 (1.14–1.28)	0.840	1.17 (0.04) 1.17 (1.09–1.29)	1.18 (0.03) 1.18 (1.13–1.28)	0.391	1.18 (0.03) 1.17 (1.13–1.28)	0.693	1.17 (0.03) 1.17 (1.09–1.29)
Chlorine-R, >1.2		11 (14.3%)	6 (27.3%)	0.199	1 (9.1%)	0.684	11 (19%)	6 (14.3%)	0.599	5 (29.4%)	0.772	17 (16.8%)
Glucose R		0.64 (0.13) 0.63 (0.45–1.38)	0.65 (0.07) 0.65 (0.51–0.84)	0.377	0.65 (0.07) 0.65 (0.51–0.75)	0.482	0.64 (0.1) 0.64 (0.44–0.84)	0.65 (0.15) 0.62 (0.45–1.38)	0.715	0.66 (0.18) 0.62 (0.45–1.38)	0.633	0.64 (0.12) 0.64 (0.44–1.38)
Glucose R, >0.65		29 (43.3%)	8 (40%)	0.804	5 (45.5%)	1.000	21 (40.4%)	16 (44.4%)	0.827	9 (40.9%)	1.000	37 (36.6%)
OCB pattern	1	10 (14.9%)	5 (26.3%)	0.123	1 (11.1%)	0.157	10 (19.6%)	6 (16.7%)	0.943	4 (18.2%)	0.793	16 (18.4%)
	2	46 (68.7%)	9 (47.4%)		5 (55.6%)		32 (62.7%)	23 (63.9%)		13 (59.1%)		55 (63.2%)
	3	11 (16.4%)	4 (21.1%)		2 (22.2%)		8 (15.7%)	7 (19.4%)		5 (22.7%)		15 (17.2%)
	4	0 (0%)	1 (5.3%)		1 (11.1%)		1 (2%)	0 (0%)		0 (0%)		1 (1%)
OCB Bands	count	13.53 (7.2) 14 (2–27)	8.4 (4.38) 7 (3–17)	**0.045**	6.8 (4.27) 5 (3–14)	**0.034**	11.19 (6.17) 10 (2–23)	14.5 (7.82) 14.5 (3–27)	0.115	16.67 (7.99) 18 (3–27)	**0.032**	12.57 (7.02) 11 (2–27)
	<7	12 (27.9%)	4 (40%)	0.485	3 (60%)	0.612	11 (35.5%)	5 (22.7%)	0.064	2 (16.7%)	**0.026**	16 (30.2%)
	7–12	10 (23.3%)	4 (40%)		1 (20%)		10 (32.3%)	4 (18.2%)		1 (8.3%)		14 (26.4%)
	12–24	17 (39.5%)	2 (20%)		1 (20%)		10 (32.3%)	9 (40.9%)		6 (50%)		19 (35.8%)
	>24	4 (9.3%)	0 (0%)		0 (0%)		0 (0%)	4 (18.2%)		3 (25%)		4 (7.5%)
IgG Index		0.88 (0.53) 0.7 (0.42–2.77)	0.64 (0.17) 0.6 (0.41–0.93)	0.115	0.6 (0.16) 0.56 (0.41–0.84)	0.114	0.71 (0.34) 0.57 (0.34–2)	0.97 (0.59) 0.81 (0.43–2.77)	**0.007**	0.96 (0.59) 0.81 (0.43–2.43)	0.140	0.82 (0.48) 0.66 (0.34–2.77)
IgG Index, >0.7		32 (48.5%))	8 (42.1%)	0.795	3 (33.3%)	0.491	18 (36%)	22 (61.1%)	**0.029**	13 (32.5%)	0.218	40 (46.5%)
25-OH vit. D, µg/L		15.17 (12.02) 12.7 (0–52.4)	11.85 (8.43) 11.35 (0.5–28.7)	0.360	9.68 (6.07) 7.7 (0.5–19.4)	0.207	14.74 (10.9) 13.1 (0.5–52.4)	13.91 (11.82) 9.3 (0–40.5)	0.573	14.91 (10.66) 17.8 (0–32.1)	0.620	14.4 (11.24) 12.7 (0–52.4)
25-OH vit. D < 20 µg/L		46 (67.6%)	17 (77.3%)	0.437	11 (100%)	**0.030**	38 (71.7%)	26 (68.4%)	0.817	15 (62.5%)	0.435	64 (70.3%)
Vit. B12, ng/L		356.48 (152.39) 333 (150–802)	294.91 (91.02) 289.5 (185–594)	0.085	289.27 (48.2) 301 (202–348)	0.267	359.34 (152.09) 330 (150–802)	315.15 (122.99) 295 (156–700)	0.135	339.24 (135.11) 317 (156–700)	0.990	341.76 (142.23) 316 (150–802)
Vit. B12 < 200 ng/L		9 (12%)	1 (4.5%)	0.447	0 (0%)	0.598	4 (6.8%)	6 (15.4%)	0.190	2 (8%)	1.000	10 (10.2%)

Sample sizes for each variable were as follows: opening pressure (n = 78), chloride ratio (n = 88), glucose ratio (n = 88), OCB type (n = 87), number of bands (n = 53), IgG index (n = 86), vitamin D (n = 91), and vitamin B12 (n = 98). Data are presented as n (%), mean (SD), or median (min–max). Statistical significance was defined as *p* < 0.05 and is highlighted in bold. VEP measurements were analyzed both as continuous (parametric) variables and as categorical variables based on predefined cut-off values for prolonged latency or reduced amplitude; however, no overlapping statistical significance was observed between the two analyses. Abbreviations: COP: cerebrospinal fluid opening pressure; Chloride-R: ratio of cerebrospinal fluid and serum chloride; Glucose-R: ratio of cerebrospinal fluid and serum glucose; OCB: oligoclonal band.

**Table 3:** Multivariable Logistic Regression Analysis. Independent predictors were identified only for any amplitude reduction.

**Amplitude Reduction, Bilateral**							**Latency Prolongation, Bilateral**					
**Variable**	**B**	**SE**	**Wald**	***p*** **-value**	**OR**	**95% CI for OR**	**B**	**SE**	**Wald**	***p*** **-value**	**OR**	**95% CI for OR**
Sex (male)	−1.352	0.749	3.262	0.071	3.866	0.891–16.769	−0.099	0.560	0.031	0.860	0.906	0.302–2.716
Disease onset age (>40)	0.319	0.844	0.143	0.705	1.376	0.263–7.194	−0.386	0.611	0.400	0.527	0.680	0.205–2.249
Pyramidal or brainstem onset	−0.529	0.772	0.470	0.493	1.697	0.374–7.706	−0.607	0.567	1.146	0.284	0.545	0.179–1.656
Infratentorial lesion(s)	−0.137	0.719	0.036	0.849	0.872	0.213–3.568	0.752	0.516	2.122	0.145	2.122	0.771–5.837
EDSS	−0.231	0.382	0.367	0.544	0.793	0.375–1.677	0.001	0.261	0.000	0.996	1.001	0.600–1.670
Vitamin D deficiency	20.089	7544	0.000	0.998	530	- *	−0.696	0.548	1.611	0.204	0.499	0.170–1.460
**Amplitude Reduction, Any**							**Latency Prolongation, Any**					
**Variable**	**B**	**SE**	**Wald**	***p*** **-value**	**OR**	**95% CI for OR**	**B**	**SE**	**Wald**	***p*** **-value**	**OR**	**95% CI for OR**
Sex (male)	1.291	0.601	4.610	**0.032**	3.635	1.119–11.808	−0.436	0.503	0.752	0.386	1.547	0.577–4.144
Disease onset age (>40)	−0.410	0.666	0.378	0.539	0.664	0.180–2.448	−0.079	0.527	0.022	0.882	0.924	0.329–2.598
Pyramidal or brainstem onset	1.764	0.641	7.562	**0.006**	5.865	1.660–20.513	−0.471	0.495	0.907	0.341	1.602	0.607–4.226
Infratentorial lesion(s)	0.101	0.560	0.033	0.857	1.106	0.369–3.319	0.111	0.458	0.059	0.808	1.118	0.455–2.745
EDSS	−0.312	0.293	1.137	0.286	0.732	0.412–1.299	−0.321	0.237	1.836	0.175	0.726	0.456–1.154
Vitamin D deficiency	0.806	0.683	1.390	0.238	2.238	0.587–8.538	−0.128	0.498	0.066	0.797	0.880	0.332–2.333

Model fit indices were as follows: bilateral amplitude reduction, χ^2^ = 12.925, df = 6, *p* = 0.044, Hosmer–Lemeshow *p* = 0.996, Nagelkerke R^2^ = 0.260; any amplitude reduction, χ^2^ = 17.182, df = 6, *p* = 0.009, Hosmer–Lemeshow *p* = 0.484, Nagelkerke R^2^ = 0.265; bilateral latency prolongation, χ^2^ = 5.761, df = 6, *p* = 0.450, Nagelkerke R^2^ = 0.093; any latency prolongation, χ^2^ = 3.872, df = 6, *p* = 0.694, Nagelkerke R^2^ = 0.058. Statistical significance was defined as *p* < 0.05 and is highlighted in bold. * Vitamin D deficiency showed convergence problems due to complete separation in the bilateral amplitude reduction model and was therefore not reliably interpretable.

## Data Availability

The datasets used and/or analysed during the current study are available from the corresponding author on reasonable request.

## Appendix A. Differences in Right- and Left-Sided VEP P100 Amplitude and Latency According to Clinical, Laboratory, and Radiological Characteristics, Reported as Mean (SD) and Median (min–max)

**Table:**

			Amplitude-R		Amplitude-L	*p*	Latency-R		Latency-L	*p*
Sex	Female		11.1 (4.96) 11.6 (1.1–28.6)	**<0.001**	13.25 (7.94) 11.85 (2.5–53)	**<0.001**	116.76 (12.92) 114 (100–180)	0.324	118.52 (14.87) 115 (100–183)	0.333
	Male		7.32 (3.26) 7.8 (1.2–15)		7.56 (4.12) 7.05 (1.9–18.7)		117.26 (9.07) 115.5 (102–137)		118.27 (9.7) 116.35 (104–145)	
Disease onset age	≥40		9.96 (4.52) 10.2 (2.5–21.2)	0.878	12.58 (9.62) 10.75 (3.4–53)	0.703	115.88 (10.61) 113.5 (100–140)	0.849	119.91 (17.17) 114.7 (103–183)	0.907
	<40		10.07 (4.98) 9.35 (1.1–28.6)		11.33 (6.66) 10.15 (1.9–36)		117.25 (12.41) 114 (101–180)		117.94 (12.2) 115.5 (100–164)	
Diagnosis	CIS		11.3 (6.47) 10.7 (4.8–28.6)	0.652	10.94 (6.42) 10 (2.3–27.2)	0.846	113.36 (5.29) 112.35 (103.8–125.1)	0.350	113.54 (6.77) 112 (105.9–127.8)	0.151
	RRMS		9.85 (4.57) 9.5 (1.1–23.7)		11.76 (7.69) 10.2 (1.9–53)		117.47 (12.61) 114 (100–180)		119.24 (14.25) 116 (100–183)	
Onset symptoms	Motor	Yes	9.52 (4.12) 9.8 (1.2–16.3)	0.833	13.85 (10.52) 11.3 (3.4–53)	0.214	116.91 (11.94) 111.9 (102–163)	0.794	117.66 (13.11) 113.4 (103–154)	0.498
		No	10.27 (5.13) 9.4 (1.1–28.6)		10.71 (5.61) 10.1 (1.9–28.3)		116.89 (12.02) 114 (100–180)		118.79 (13.87) 116 (100–183)	
	Sensory	Yes	10.58 (4.11) 11.05 (1.1–21.2)	**0.046**	12.71 (8.42) 11.4 (1.9–53)	**0.044**	116.89 (12.43) 114 (100–180)	0.924	118.03 (14.08) 115 (100–183)	0.646
		No	9.35 (5.61) 7.5 (1.2–28.6)		10.3 (5.98) 8.9 (3.3–27.2)		116.9 (11.41) 114 (101–163)		118.98 (13.07) 115.75 (103–154)	
	Brainstem	Yes	8.36 (4.95) 7.9 (1.2–21.2)	**0.048**	9.83 (10.03) 7.2 (2.3–53)	**0.003**	120.91 (13.13) 118.5 (101–163)	**0.031**	122.78 (12.12) 120.5 (103–154)	**0.003**
		No	10.57 (4.72) 10.65 (1.1–28.6)		12.23 (6.5) 11 (1.9–36)		115.65 (11.34) 112 (100–180)		117.1 (13.81) 113 (100–183)	
	Other	Yes	11.48 (6.42) 11.3 (1.2–28.6)	0.318	11.93 (6.39) 9.1 (4.1–27.2)	0.870	118.28 (12.86) 117 (103.8–163)	0.671	118.78 (12.44) 116.1 (105–154)	0.527
		No	9.61 (4.22) 9.5 (1.1–21.2)		11.57 (7.85) 10.5 (1.9–53)		116.49 (11.71) 113.5 (100–180)		118.35 (13.98) 115 (100–183)	
	Pyramidal or BS	Yes	9.08 (4.56) 9 (1.2–21.2)	0.113	11.53 (9.2) 9.8 (2.3–53)	0.182	117.45 (10.96) 114.6 (101–163)	0.392	118.66 (11.74) 116 (103–154)	0.359
		No	10.93 (4.97) 11.1 (1.1–28.6)		11.77 (5.61) 10.8 (1.9–28.3)		116.38 (12.87) 112.9 (100–180)		118.25 (15.24) 114.5 (100–183)	
Initial EDSS	≥3		10.06 (4.59) 11.3 (3.2–16.3)	0.731	14.61 (14.93) 10 (3.3–53)	0.838	115.67 (8.77) 112 (108–137)	0.821	117.06 (9.68) 113 (111–142)	0.995
	<3		10.04 (4.89) 9.3 (1.1–28.6)		11.36 (6.43) 10.2 (1.9–36)		117.02 (12.23) 114 (100–180)		118.58 (13.94) 116 (100–183)	
Periventricular	Yes		9.63 (4.33) 9.2 (1.1–23.7)	0.058	10.74 (5.67) 10 (1.9–35)	0.165	117.17 (12.49) 114 (100–180)	0.516	118.6 (14.11) 115.5 (100–183)	0.832
	No		12.9 (6.17) 14.35 (3.2–21.2)		14.55 (9.96) 13.1 (3.6–36)		114.56 (8.45) 111.15 (108–133.2)		115.53 (6.75) 113.25 (109.2–129)	
Cortical/juxtacortical	Yes		10.12 (4.53) 10 (1.1–23.7)	0.527	11.27 (6.4) 10.45 (2.3–36)	0.576	117.21 (11.25) 114 (101–163)	0.762	117.96 (12.42) 115 (100–154)	0.624
	No		9.52 (4.68) 9.2 (1.2–21.2)		10.66 (5.74) 9.1 (1.9–28.3)		116.48 (13.93) 114 (100–180)		119.05 (15.83) 116 (103–183)	
Corpus callosum	Yes		9.12 (3.92) 8.6 (1.1–19.2)	0.140	10.8 (6.61) 9 (2.5–35)	0.437	117.51 (10.8) 114 (101–140)	0.514	119.43 (13.91) 116 (100–150)	0.639
	No		10.47 (4.77) 11 (1.2–23.7)		11.28 (5.97) 10.5 (1.9–36)		116.51 (12.99) 114 (100–180)		117.74 (13.7) 114.5 (103–183)	
Infratentorial	Yes		9.42 (4.28) 8.75 (2–21.2)	0.316	10.24 (5.94) 9.65 (3.3–35)	0.131	118.12 (9.93) 115 (103–144)	0.104	119.23 (11.82) 116 (104–150)	0.224
	No		10.27 (4.77) 11 (1.1–23.7)		11.65 (6.29) 10.9 (1.9–36)		116.09 (13.67) 112 (100–180)		117.69 (14.95) 113.4 (100–183)	
Cervical	Yes		9.89 (4.78) 9.25 (1.1–23.7)	0.877	11.5 (6.82) 10.15 (2.5–36)	0.757	117.95 (14.2) 113.5 (100–180)	0.575	119.76 (16.02) 116 (100–183)	0.321
	No		9.79 (4.47) 10 (2.9–17.8)		10.35 (5.31) 10 (1.9–22.8)		114.41 (7.05) 114 (102–138)		115.4 (9.08) 113.2 (104–150)	
Thoracic	Yes		7.87 (4.92) 6.65 (1.2–17.8)	0.051	9.88 (8) 7.9 (1.9–35)	0.186	120.64 (15.33) 115 (101–163)	0.067	122.84 (14.78) 121.35 (103–154)	0.087
	No		10.86 (4.28) 11.9 (1.1–17.8)		11.69 (5.97) 10.55 (2.5–28.3)		114.89 (16.35) 111.15 (100–180)		117.99 (20.26) 111 (100–183)	
COP, cmH_2_O	>20		8.15 (2.34) 7.9 (4.8–13.1)	0.308	11.41 (7.49) 9.8 (2.3–28.3)	0.983	116.91 (8.76) 118 (100–133)	0.605	125.47 (22.35) 117 (105–183)	0.468
	<20		9.9 (5.24) 10.3 (1.1–28.6)		11.01 (6.56) 10.3 (1.9–36)		118.09 (13.37) 114 (101–180)		118.76 (12.45) 116 (100–164)	
Protein, mg/dL	>40		8.15 (3.94) 8.5 (1.2–17.8)	**0.047**	9.3 (4.05) 9.8 (1.9–17.9)	0.180	119.45 (13.99) 116 (103.8–163)	0.421	122.13 (14.93) 119 (104.4–154)	0.223
	<40		10.55 (4.96) 10.6 (1.1–28.6)		12.28 (8.09) 10.7 (2.3–53)		116.23 (11.34) 113.5 (100–180)		117.48 (13.14) 114.5 (100–183)	
Cell,/mm3	>10		10.85 (3.4) 11.6 (3.8–15.9)	0.305	11.31 (3.92) 11.7 (1.9–19.4)	0.354	116.25 (9.68) 114 (107–144)	0.885	118.11 (10.37) 116 (103–145)	0.692
	<10		9.94 (5.07) 9.2 (1.1–28.6)		11.73 (8.04) 10 (2.3–53)		117.08 (12.39) 114 (100–180)		118.58 (14.19) 115.5 (100–183)	
Chlorine-R,	>1.2		8.68 (4.23) 8.9 (1.1–17.8)	0.316	11.09 (8.63) 9.1 (2.5–35)	0.284	119.85 (21.75) 111.9 (100–180)	0.497	123.46 (24.18) 111 (100–183)	0.588
	<1.2		10.3 (4.96) 9.8 (2–28.6)		11.75 (7.36) 10.45 (1.9–53)		116.36 (8.88) 114 (101–144)		117.5 (10.22) 116 (103–150)	
Glucose R,	>0.65		10.46 (5.58) 10 (2.5–28.6)	0.554	12.34 (6.76) 12 (1.9–36)	0.095	116.64 (11.95) 114 (100–163)	0.916	117.88 (12.61) 116 (103–164)	0.866
	<0.65		9.53 (3.95) 9.4 (1.1–21.2)		10.3 (5.95) 9.85 (2.3–35)		116.74 (12.83) 113 (102–180)		119.04 (14.83) 115 (100–183)	
OCB type	1		9.3 ± 4.2 8.3 (2.5–16.2)	0.164	9.6 ± 4.5 9 (3.6–16.5)	**0.024** *	113.6 ± 7 111.6 (103.8–125.1)	0.637	115.4 ± 11.6 110.1 (103–144)	0.544
	2		11.2 ± 5.2 11 (1.1–28.6)		13.8 ± 8.9 12.2 (2.5–53)		117 ± 12.8 114 (100–180)		118.8 ± 14.6 116 (100–183)	
	3		8.4 ± 3.9 9.2 (1.2–13.5)		8.8 ± 3.1 9.8 (1.9–13.6)		116.4 ± 8 114 (106–133)		118.3 ± 9.6 113 (106–137)	
	4		5.6 ± 0 5.6 (5.6–5.6)		2.3 ± 0 2.3 (2.3–2.3)		118 ± 0 118 (118–118)		117 ± 0 117 (117–117)	
OCB Bands	<7		10.8 ± 6.4 8.7 (3.2–28.6)	0.195	11.1 ± 6.4 9.4 (4.1–27.2)	0.244	113.5 ± 5.9 114 (101–125.1)	0.363	115.5 ± 10 115.3 (103–147.9)	0.107
	7–12		8.8 ± 4.4 8.9 (2.5–19.2)		18.1 ± 13.4 13 (5.2–53)		115.2 ± 8.2 113.8 (100–135)		120.3 ± 19.2 115.5 (105–183)	
	12–24		12.1 ± 5.2 12 (1.1–23.7)		13.4 ± 7.1 12.2 (2.5–36)		116.2 ± 11.1 112 (101–140)		116.8 ± 12.1 115 (100–145)	
	>24		11.7 ± 1.2 12 (10–12.7)		9.9 ± 1.7 9.8 (8.4–11.7)		126.5 ± 14.5 125.5 (111–144)		125.5 ± 5.9 125 (120–132)	
IgG index	>0.7		10.51 (4.67) 10.3 (1.2–23.7)	0.613	11.21 (6.11) 10.6 (1.9–36)	0.558	118.88 (13.68) 115 (105–180)	0.144	120.11 (12.67) 117 (104.4–164)	0.078
	<		10.08 (5.18) 9.3 (1.1–28.6)		12.85 (9.19) 10.6 (2.3–53)		114.14 (7.92) 112 (100–135)		116.5 (13.59) 114.2 (100–183)	
25-OH vit.Dµg/L	<20		9.49 (4.54) 9.2 (1.1–19.2)	0.871	10.95 (6.27) 10 (1.9–35)	0.745	116.49 (13.14) 114 (100–180)	0.255	118.94 (15.18) 115.75 (100–183)	0.587
	>		9.55 (4.25) 8.8 (2–23.7)		10.71 (6.4) 10.1 (3.6–36)		118.46 (9.97) 112.8 (105–144)		118.59 (11.09) 116 (105–150)	
Vit. B12 ng/L	<200		9.37 (4.03) 9.2 (1.1–19.2)	0.108	11.17 (7.47) 10 (1.9–53)	0.174	115.88 (9.06) 113.5 (101–144)	0.879	116.96 (10.7) 115 (100–150)	0.318
	>		12.35 (6.05) 12.4 (2.5–23.7)		13.73 (7.03) 12.1 (6.6–28.3)		123.4 (26.44) 114.5 (100–180)		129.8 (27.47) 118 (105–183)	

Bolded rows indicate characteristics that remained statistically significant when VEP parameters were additionally analyzed as categorical outcomes (i.e., amplitude reduction or latency prolongation). * Post hoc pairwise comparisons with Bonferroni correction revealed no statistically significant differences between the groups (all adjusted *p*-values > 0.05).
